# Potential contributions of an on-site nurse mentoring program on neonatal mortality reductions in rural Karnataka state, South India: evidence from repeat community cross-sectional surveys

**DOI:** 10.1186/s12884-020-02942-8

**Published:** 2020-04-23

**Authors:** Ramesh Banadakoppa Manjappa, Arin Kar, Krishnamurthy Jayanna, Jyothi S. Hallad, Troy Cunningham, Rajaram Potty, H. L. Mohan, Maryanne Crockett, Janet Bradley, Elizabeth Fischer, H. Sudarshan, James F. Blanchard, Stephen Moses, Lisa Avery

**Affiliations:** 1 Centre for Global Public Health, Department of Community Health Sciences, University of Manitoba, R070 Med Rehab Bldg, 771 McDermot Avenue, Winnipeg, Manitoba R3E 0T6 Canada; 2 Karnataka Health Promotion Trust, Bangalore, India; 3 JSS Institute of Economic Research, Dharwad, India; 4 IntraHealth International, Chapel Hill, USA; 5 Karuna Trust, Bangalore, India

**Keywords:** Nurse mentoring, Quality of care, Neonatal mortality

## Abstract

**Background:**

We assessed the effects of a nurse mentoring program on neonatal mortality in eight districts in India.

**Methods:**

From 2012 to 2015, nurse mentors supported improvements in critical MNCH-related practices among health providers at primary health centres (PHCs) in northern Karnataka, South India. Baseline (*n* = 5240) and endline (*n* = 5154) surveys of randomly selected ever-married women were conducted. Neonatal mortality rates (NMR) among the last live-born children in the three years prior to each survey delivered in NM and non-NM-supported facilities were calculated and compared using survival analysis and cumulative hazard function. Mortality rates on days 1, 2–7 and 8–28 post-partum were compared. Cox survival regression analysis measured the adjusted effect on neonatal mortality of delivering in a nurse mentor supported facility.

**Results:**

Overall, neonatal mortality rate in the three years preceding the baseline and endline surveys was 30.5 (95% CI 24.3–38.4) and 21.6 (95% CI 16.3–28.7) respectively. There was a substantial decline in neonatal mortality between the survey rounds among children delivered in PHCs supported by NM: 29.4 (95% CI 18.1–47.5) vs. 9.3 (95% CI 3.9–22.3) (*p* = 0.09). No significant declines in neonatal mortality rate were observed among children delivered in other facilities or at home. In regression analysis, among children born in nurse mentor supported facilities, the estimated hazard ratio at endline was significantly lower compared with baseline (HR: 0.23, 95% CI: 0.06–0.82, *p* = 0.02).

**Conclusion:**

The nurse mentoring program was associated with a substantial reduction in neonatal mortality. Further research is warranted to delineate whether this may be an effective strategy for reducing NMR in resource-poor settings.

## Background

Globally, the decline in neonatal mortality observed over the last several years has been slower than the decline in mortality for older children (1–59 months) [1]. Achieving faster reductions in neonatal mortality has therefore been a key area of effort for the global health community. The decline in neonatal mortality in India has also been slow [2]. Nearly three-quarters of infant deaths in India occur within 28 days of birth, and with a neonatal mortality rate of 29 per 1000 in 2015, India accounts for almost one-fourth of the estimated 2.8 million newborn deaths globally [3, 4]. Furthermore, one-third of neonatal deaths occurs on the first day of life, when newborns are often still in health facilities [5]. Karnataka state, South India, one of the better performing Indian states with respect to neonatal mortality, experienced a greater decline in neonatal mortality from 2000 to 2011 than all of India (40/1000 to 24/1000 versus 44/1000 to 31/1000) [6]. Within Karnataka however, there are large geographical disparities in health outcomes, particularly between the north and south [7, 8]. In 2012, eight districts in northern Karnataka were designated as high priority by the Government of India’s National Rural Health Mission (NRHM), based on a composite health index [9].

In October 2011, the University of Manitoba and the Karnataka Health Promotion Trust, with other partners, initiated a technical assistance project to improve maternal, newborn and child health (MNCH) outcomes through the NRHM in eight northern districts of Karnataka. The goal was to improve MNCH outcomes through the development and adoption of effective operational and health system approaches within the NRHM umbrella. One key project approach was the development of a nurse mentoring (NM) program. We introduced on-site peer mentoring of staff nurses (SN) and auxiliary nurse midwives (ANM) responsible for labour, delivery and post-partum care in primary health centres (PHC), where 24% of deliveries occurred. The program focused on: enhancing clinical skills and practices; improving quality of care through team collaboration and problem solving; and supporting initiatives to address process-related issues, including improved infrastructure, supplies and referral logistics.

We have previously demonstrated that nurse mentoring improves the MNCH-related knowledge and skills of providers in PHCs, as well as institutional readiness to provide quality care [10, 11]. These studies reported the findings of a cluster randomized trial involving all functional 24/7 PHCs in two districts of Karnataka. We used a parallel, cluster randomized trial design in which 54 of 108 facilities received six onsite mentoring visits, along with an initial training update and additional training and support in the use of case sheets for labour and delivery. Pre- and post-intervention surveys using facility audits, provider interviews and case sheet audits indicated that: (1) a higher number of facilities in the intervention arm had all appropriate drugs, equipment and supplies to deal with maternal and newborn complications; (2) the providers in the intervention arm had better knowledge of labour and delivery care, including neonatal resuscitation and low birth weight newborn care; and (3) the providers in the intervention arm showed greater compliance with the protocols during labour monitoring, delivery, and immediate post-partum care for mothers and newborns. The nurse mentoring model was subsequently scaled up in all the control PHCs in these two districts, as well as in all the PHCs in 6 other neighbouring districts. The cluster randomized trial reported earlier did not collect data on neonatal mortality. Data on the impact of quality improvement efforts in improving outcomes is limited [12, 13], and the impact on neonatal mortality is rarely measured [14]. In this paper, we assess the potential contribution of the NM program on neonatal mortality reductions.

## Methods

### The intervention

Of the 387 rural PHCs in the 8 districts that are open at all hours (24 × 7 PHCs), 385 were included in the NM program (two were not functioning during the study period). Typically, the 24 × 7 PHCs in the project districts had 2–3 Staff Nurses, two Medical Officers, one Lab Technician, one Pharmacist, one Office Assistant and around 3–4 Staff responsible for housekeeping. These facilities were supported by 53 nurse mentors, each responsible for 6–7 PHCs. The mentors had a basic qualification in general nurse midwifery (GNM), had no or little prior experience in conducting deliveries and were recruited locally. The nurse mentors were trained for five weeks in: essential clinical competencies; how to effectively mentor PHC staff; team building and collaborative problem solving; and service delivery improvement. Nurse mentors worked in teams of two and visited assigned PHCs for 2–3 days every two months initially and then quarterly for a total of six visits per year, over a period of 16–28 months. For continuity, the same nurse mentor team was assigned to the same 6–7 PHCs over the duration of the intervention. Self-assessments, observations, clinical audits, modeling of good practice, and bed-side and small group teachings were used to address gaps in: 1) clinical competencies, specifically identification and initial management of MNCH complications, including essential newborn care; 2) procurement of essential supplies; and 3) facility preparedness and referral systems. In order to improve newborn outcomes, the PHC staff were mentored to better manage birth asphyxia; to improve the availability of equipment to clear airways effectively, including bag and mask ventilators; improve the ability to provide positive pressure ventilation; and improve newborn care, particularly drying and swaddling the babies, and early initiation of breastfeeding and skin to skin contact. The mentors themselves did not perform all resuscitation, but assisted the staff nurse. A more detailed description of nurse mentor visit and what they did can be found in one of our previous publications [15]. The nurse mentors received about one and a half times more remuneration than the staff nurses in these facilities. The intervention costed an additional $5.60 per delivery [10].

### Conceptual framework

The factors associated with neonatal mortality were grouped into four broad categories:

1. maternal socio-demographic characteristics: age at the time of birth; literacy; employment; caste/tribe; religion; and socio-economic status (wealth quintiles);
2. pregnancy characteristics: pregnancy order; presence of first antenatal care (ANC) visit in the first trimester; number of ANC visits; whether received two tetanus toxoid (TT) injections; consumption of 100 iron and folic acid (IFA) tables; presence of any pregnancy complication; frequency of frontline community health worker interaction (with accredited social health activist [ASHA]); and transportation used to reach delivery site;
3. delivery characteristics: birth order; sex of child; delivery outcome (single/multiple); delivery place; delivery type (normal/Caesarian-section/assisted); delivery complication; and birth weight; and
4. post-delivery characteristics: use of postnatal care at home or at facility; and stay at the facility for at least 48 h.

Information on these factors was collected from household-based cross-sectional surveys that were conducted before and after program implementation. Data were collected on the last birth during the three years prior to each survey.

### The surveys

Baseline (March–June 2012) and endline (April–June 2015) cross-sectional surveys were carried out. A two-stage systematic stratified sampling methodology was used, with selection of villages, followed by households and eligible women. In the first stage of each round, 167 of the 5190 villages were selected using probability proportional to population size sampling. Villages were stratified according to sub-districts and village size before selection. In each survey round, lists of households with at least one eligible woman, defined as ever-married woman aged 15–34 years, were prepared separately for the Scheduled Caste (SC) or Scheduled Tribe (ST) households, and for non-SC/ST households; these lists were used for the selection of households in the second stage. From each selected village, 30 households (15 each from the SC/ST and non-SC/ST lists) were sampled by systematic sampling with equal probability, and without replacement. “Scheduled caste” is the officially designated group in India to denote lower castes in the traditional Indian social hierarchy based on caste. A large body of data exists in India to show that caste and religion play an important role in health outcomes including access and utilization of health services. All eligible women within a selected household were interviewed. A total of 5240 and 5154 ever-married women aged 15–34 years were interviewed in the baseline and endline surveys, respectively. The surveys were implemented by the Population Research Centre, Dharwad, an external, independent agency.

### Statistical analyses

The primary study outcome variable was neonatal death, defined as any death of a live-born child during the first 28 completed days of life. Baseline and endline data were merged to form one database to allow analyses across periods, and Stata 14 (StataCorp, Texas, USA) was used for analysis. Baseline and endline neonatal mortality rate (NMR) among the last live-born children in the three-years prior to each survey were computed for the following three groups, according to place of delivery: (1) PHCs supported by an NM program; (2) sub-centres, community health centres, sub-district hospitals, district hospitals and private facilities not supported by an NM program; and (3) home deliveries. Mortality rates on day 1 post-partum, days 2–7, and days 8–28 were estimated separately for each delivery group. Survey data on date of birth, whether the child survived, and the date of death for those not surviving, were used to compute the probabilities that the newborn died within 28 days of birth. The unadjusted hazard ratios (HRs) were computed using Cox survival regression [16].

We assessed whether the samples across different delivery sites, geographical locations and population groups differed in terms of known risk factors for neonatal mortality between the two rounds, using the Pearson χ^2^ test. We also explored whether these factors were associated with the differences in the probability of dying within 28 days of birth between the survey rounds, in each of the place-of-delivery-groups, using multivariate Cox regression.

## Results

Table 1 presents the distribution of births, estimated NMR and HRs by place of delivery. Overall, 24% of the births at baseline and endline occurred in PHCs supported by NM. Another 50% of births at baseline and 59% at endline were in non-NM supported facilities. The remaining 26 and 18% of deliveries, at baseline and endline respectively, occurred at home. While there was no significant change in the share of PHC deliveries, the share of home deliveries decreased between survey rounds.

**Table 1:** Distribution of samples, neonatal mortality estimates and un-adjusted hazard ratios, by place of delivery

	Deliveries in nurse mentor supported facilities		Deliveries in facilities not supported by nurse mentors		Home deliveries		Total deliveries	
	Baseline	Endline	Baseline	Endline	Baseline	Endline	Baseline	Endline
N (%)	546 (23.8)	536 (23.7)	1185 (50.1)	1280 (58.6)	600 (26.2)	363 (17.6)	2331 (100.0)	2179 (100.0)
NMR [95% CI]	29.4 [18.1–47.5]	9.3 [3.9–22·3]	32.2 [23·5–44.0]	25.8 [18.4–36.1]	28.3 [17.7–45.2]	24.9 [13.0–47.3]	30.5 [24.3–38.4]	21.6 [16.3–28.7]
Hazard ratio [95% CI] p	Ref.	0.36 [0.11–1.16] 0.09	Ref.	0.61 [0.27–1.35] 0.22	Ref.	1.04 [0.43–2.54] 0.93	Ref.	0.64 [0.37–1.12] 0.12

There were no statistically significant differences in neonatal mortality among the three place of delivery groups at baseline (*p* = 0.90). The estimated NMR among the 2331 and 2179 last-born children in the three years preceding the baseline and endline surveys were 30.5 (95% CI 24.3–38.4) and 21.6 (95% CI 16.3–28.7) per 1000 live births. This overall declining trend was not statistically significant (HR 0.64, 95% CI 0.37–1.12, *p* = 0.12). There was a substantial decline however in neonatal mortality between the survey rounds among children delivered in PHCs supported by NM: HRs of 29.4 (95% CI 18.1–47.5) vs. 9.3 (95% CI 3.9–22.3), *p* = 0·09. There were no significant declines in NMR among children delivered in non-NM facilities or at home.

Table 2 presents the NMR estimates and HRs for different periods of time after birth by place of delivery. The maximum NMR reductions between baseline and endline are seen on day 1 of birth in NM-supported facilities (12.8 at baseline to 1.9 at endline; HR 0.10, 95% CI 0.01–0.90, *p* = 0.04). Differences in NMR after day 1 were not statistically significant for deliveries in non-NM facilities or at home. There were no reported deaths among those delivered in NM-supported facilities during days 8–28 post birth, compared to NMRs of 4 and 8.5 among those delivered in unsupported facilities or at home. Mortality during days 8–28 after birth went up significantly at endline for home deliveries.

**Table 2:** Neonatal mortality estimates and unadjusted hazard ratios for different periods since birth, by place of delivery

Mortality rates for periods since birth	Deliveries in nurse mentor supported facilities		Deliveries in facilities not supported by nurse mentors		Home deliveries		Total deliveries	
	Baseline	Endline	Baseline	Endline	Baseline	Endline	Baseline	Endline
**Day 1** NMR [95% CI]	12.8 [6.1–26.7]	1.9 [0.3–13.2]	4.2 [1.8–10.1]	6.2 [3.1–12.5]	10.0 [4.5–22.1]	5.5 [1.4–21.8]	7.7 [4.9–12.2]	5.0 [2.8–9.1]
Hazard ratio [95% CI] p	Ref	0.10 [0.01–0.90] 0.04	Ref.	0.99 [0.28–3.52] 0.99	Ref.	0.44 [0.08–2.45] 0.44	Ref.	0.58 [0.24–1.42] 0.23
**Days 2–7** NMR [95% CI]	11.1 [5.0–24.6]	7.5 [2.8–19.8]	21.2 [14.4–31.3]	15.7 [10.2–24.3]	16.8 [9.1–31.1]	11.1 [4.2–29.3]	17.7 [13.1–24.0]	12.9 [8.9–18.7]
Hazard ratio [95% CI] p	Ref	0.98 [0.24–4.02] 0.97	Ref	0.50 [0.17–1.43] 0.20	Ref	0.58 [0.15–2.30] 0.44	Ref	0.59 [0.26–1.33] 0.20
**Days 8-28** NMR [95% CI]	5.7 [1.8–17.5]	0.0 [NC]	7.0 [3.5–14.0]	4.0 [1.7–9.6]	1.7 [0.2–12.1]	8.5 [2.7–26.1]	5.3 [3.0–9.4]	3.8 [1.9–7.5]
Hazard ratio [95% CI] p	NC		Ref	0.79 [0.18–3.49] 0.76	Ref	42.05 [4.42–399.76] 0.00	Ref	0.96 [0.33–2.84] 0.94

*NC* Not computed

As the PHCs were not randomized for the intervention, we examined changes between survey rounds in factors that determine the choice of facility for delivery, which may confound the effects of NM on neonatal mortality. The socio-demographic profiles of women in each delivery-place type did not differ significantly between the two survey rounds. Although a greater proportion of literate women, women engaged in housework, non-SC/ST women, and women in higher wealth index quintiles were more likely to deliver in a non-NM facility (data not shown), this pattern did not change between the two surveys. Additionally, between survey rounds, there were no significant differences in the overall sample profiles based on these socio-demographic characteristics. Hence, the socio-demographic differences in the choice of place of delivery likely do not account for the change in estimated NMR between surveys across delivery sites.

Table 3 examines the changes in known risk factors associated with neonatal mortality between the two survey rounds, according to delivery place. There were no significant differences in socio-demographic characteristics. However, there were significant differences between baseline and endline with respect to pregnancy characteristics. These included increases in: timing of the first ANC visit; number of ANC checkups; coverage with two TT injections; consumption of 100 IFA tablets during pregnancy; frequency of contact with an ASHA; and use of ambulance transport to reach the delivery point. There was also a decline in reported complications during pregnancy. Similar trends for these characteristics were observed among the two groups who delivered in NM- supported and unsupported facilities. The differences between survey rounds for home deliveries were significant only with regard to IFA consumption, frequency of ASHA meetings and reporting of pregnancy complications.

**Table 3:** Population and health care characteristics according to place of delivery, at baseline and endline

Characteristic	Deliveries in nurse mentor supported facilities			Deliveries in facilities not supported by nurse mentors			Home deliveries			Total deliveries		
	Base-line	End-line	P	Base-line	End-line	p	Base-line	End-line	p	Base-line	End-line	p
**Maternal age**												
< 20 years	31.1	32.4	0.75	31.5	30.7	0.77	22.4	31.0	0.01	29.0	31.1	0.30
20+ years	68.9	67.7		68.6	69.3		77.6	39.3		71.0	68.9	
**Maternal literacy**												
Literate	47.5	47.9	0.93	58.9	60.9	0.47	28.0	30.0	0.60	48.1	52.4	0.09
Illiterate	52.5	52.1		41.2	39.1		72.0	70.0		51.9	47.6	
**Maternal work**												
Housework	51.1	44.8	0.14	58.7	59.0	0.94	39.5	34.3	0.24	51.9	51.3	0.80
Other	48.9	55.2		41.3	41.0		60.5	65.7		48.1	48.7	
**Caste/tribe**												
Scheduled caste/tribe	35.1	42.9	0.24	31.8	36.0	0.37	41.6	50.1	0.23	35.2	40.1	0.29
Other	64.9	57.1		68.2	64.0		58.4	49.9		64.8	59.9	
**Religion**												
Hindu	88.6	92.6	0.13	89.1	88.8	0.91	90.2	91.4	0.68	89.3	90.2	0.63
Other	11.4	7.4		10.9	11.2		9.8	5.6		10.7	9.8	
**Wealth index quintiles**												
4–5	32.7	29.3	0.42	26.3	21.7	0.11	40.5	43.0	0.72	31.6	27.3	0.11
3	31.3	35.9		29.2	33.3		34.0	30.9		30.9	33.5	
1–2	36.0	34.8		44.5	45.0		25.5	26.1		37.5	39.3	
**First ANC**												
In 1st trimester	67.0	75.5	0.03	70.4	77.1	0.02	50.8	57.3	0.13	64.4	73.2	< 0.01
Later/never	33.0	24.5		29.6	22.9		49.2	42.7		35.6	26.8	
**# of ANC checkups**												
3 or more	77.9	87.1	< 0.01	86.1	90.3	0.01	64.1	69.2	0.18	78.4	85.8	< 0.01
< 3 or none	22.1	12.9		13.9	9.7		35.9	30.8		21.6	14.2	
**2 TT injections**												
Yes	90.1	93.9	0.17	9.0	95.5	0.01	83.5	85.6	0.50	89.3	93.4	< 0.01
No	9.9	6.1		8.0	4.5		16.5	14.4		10.7	6.6	
**Consumed 100 IFA tablets**												
Yes	14.0	24.2	< 0.01	17.8	28.8	< 0.01	10.0	18.7	< 0.01	14.9	25.9	< 0.01
No	86.0	75.8		82.2	71.2		90.0	81.3		85.1	74.1	
**ASHA**^**1**^**meeting frequency (ANC)**												
At least once a month	44.4	55.0	0.03	41.6	52.5	< 0.01	34.4	43.5	0.02	40.4	51.5	< 0.01
Once in 3 months/ less	55.6	45.0		58.4	47.5		65.6	56.5		59.6	48.5	
**Pregnancy complication**^**2**^												
Any	50.6	43.4	0.06	58.3	48.3	< 0.01	47.7	36.2	0.01	53.7	45.0	< 0.01
None	49.4	56.6		41.7	51.7		52.3	63.8		46.3	55.0	
**Transportation to delivery facility**												
Ambulance	23.6	31.9	0.02	27.8	33.6	0.04	NA	NA	NA	26.4	33.1	0.01
Other	76.4	68.1		72.2	66.4					73.6	66.9	
**Delivery type**												
Normal	98.7	99.4	0.32	80.1	77.6	0.30	99.8	99.7	0.74	89.6	86.7	0.03
Other^3^	1.3	0.6		19.9	22.4		0.2	0.3		10.4	13.3	
**Delivery outcome**												
Single	100.0	98.8	< 0.01	96.6	97.2	0.71	98.8	99.2	0.71	98.0	97.9	0.93
Multiple	0.0	1.2		3.4	2.8		1.2	0.8		2.0	2.1	
**Child’s sex**												
Male	50.8	49.9	0.82	51.2	52.9	0.51	55.2	49.1	0.16	52.2	51.5	0.77
Female	49.2	50.1		48.8	47.1		44.8	50.9		47.8	48.5	
**Delivery complication**^**4**^												
Any	20.5	13.3	0.03	24.5	23.1	0.56	11.5	10.7	0.76	20.1	18.6	0.39
None	79.5	86.7		75.6	76.9		88.5	89.3		79.9	81.5	
**Birth weight**												
< 2500 g	11.8	13.0	0.64	14.5	16.3	0.32	6.0	5.5	0.24	11.6	14.1	0.06
Other	88.2	87.0		85.5	83.7		94.0	91.5		88.4	85.6	
**Postnatal care either at home or at facility**												
Yes	54.6	84.8	< 0.01	59.2	89.2	< 0.01	39.4	42.1	0.57	52.9	79.8	< 0.01
No	45.4	15.3		40.8	10.8		60.6	57.9		47.1	20.2	
**48 h facility stay post delivery**												
Yes	29.6	34.5	0.26	53.7	61.9	0.01	NA	NA	NA	45.9	54.0	< 0.01
No	70.4	65.5		46.3	38.1					54.1	46.0	

^1^Accredited Social Health Activist

^2^Swelling of hands/feet and face, excessive vomiting/giddiness, headache/visual disturbances, weakness/excessive fatigue, convulsions not with fever, weak or no movement of fetus, abnormal position of fetus, jaundice, vaginal bleeding, vaginal discharge, malaria, abdominal pain, preterm/premature rupture of membrane, preterm labour

^3^Includes Caesarean section and assisted deliveries

^4^Premature labour, preterm/premature rupture of membrane, prolonged labour (> 12 h), obstructed labour, breech/malpresentation

*NA* Not applicable

Except for an increase in the proportion having Caesarean section or assisted deliveries (from 10 to 13%), there were no significant differences overall between baseline and endline according to delivery characteristics. In NM facilities there was a significant increase between the rounds in the proportion of multiple births (from 0 to 1%). In NM-supported facilities, a significantly lower proportion of women at endline reported delivery complications (13%) compared to baseline (21%). None of the differences in delivery characteristics between the two survey rounds were statistically significant for those deliveries in a non-NM facility or at home. In all facilities, there was a significant improvement in the proportion of women who received post natal care, either at home or in a facility (53 to 80%). Similarly, there was a significant increase in the overall proportion of women who stayed at facilities for at least 48 h post delivery, from 46% at baseline to 54% at endline.

Table 4 presents the results of the Cox multivariate regression analysis, with the probability of a child dying within 28 days of birth according to place of delivery as the primary outcome, and the selected risk factors as predictors, controlling for survey round. Only risk factors that differed significantly (*p* < 0.05) between the survey rounds in any of the four survey groups analyzed in Table 3 were included in the multivariate regression model. For deliveries in NM facilities, delivery type and type of gestation were excluded from the model due to small numbers. Similarly, the model for the home deliveries excluded delivery type, transportation to delivery point and 48-h stay in the facility. The model for total deliveries included delivery place and an interaction term involving delivery place and survey round. Overall, single births and births to mothers who had received two TT injections during pregnancy were associated with increased survival of the neonate, when the survey round and place of delivery were controlled for. The risk of dying within 28 days of birth was significantly greater for neonates born to mothers who used an ambulance to reach the delivery point. Neither the survey round nor the interaction between survey round and delivery place were significantly and independently associated with neonatal mortality in the total sample.

**Table 4:** Results of the Cox multivariate regression analyses – probability of death within 28 days

Characteristic	Deliveries in nurse mentor supported facilities			Deliveries in facilities not supported by nurse mentors			Home deliveries			Total deliveries		
	HR	95% CI	p	HR	95% CI	p	HR	95% CI	p	HR	95% CI	p
**Survey round**												
Baseline	Ref.	Ref.	Ref.	Ref.	Ref.	Ref.	Ref.	Ref.	Ref.	Ref.	Ref.	Ref.
Endline	0.23	0.06–0.82	0.02	0.67	0.37–1.22	0.19	1.23	0.48–3.19	0.67	1.05	0.41–2.69	0.91
**First ANC**												
In 1st trimester	0.70	0.21–2.29	0.56	1.78	0.84–3.77	0.13	0.99	0.31–3.18	0.99	1.42	0.78–2.60	0.25
Later/never	Ref.	Ref.	Ref.	Ref.	Ref.	Ref.	Ref.	Ref.	Ref.	Ref.	Ref.	Ref.
**# of ANC checkups**												
3 or more	0.38	0.07–1.95	0.25	1.90	0.58–6.25	0.29	2.50	0.55–11.32	0.23	1.86	0.73–4.73	0.19
< 3 or none	Ref.	Ref.	Ref.	Ref.	Ref.	Ref.	Ref.	Ref.	Ref.	Ref.	Ref.	Ref.
**2 TT injections**												
Yes	1.72	0.23–13.09	0.60	0.22	0.10–0.47	< 0.01	1.78	0.28–11.20	0.54	0.34	0.13–0.83	0.02
No	Ref.	Ref.	Ref.	Ref.	Ref.	Ref.	Ref.	Ref.	Ref.	Ref.	Ref.	Ref.
**Consumed 100 IFA tablets**												
Yes	4.53	1.40–14.69	0.01	1.62	0.91–2.91	0.10	0.34	0.03–3.86	0.38	1.33	0.80–2.20	0.27
No	Ref.	Ref.	Ref.	Ref.	Ref.	Ref.	Ref.	Ref.	Ref.	Ref.	Ref.	Ref.
**ASHA meeting frequency (ANC)**												
At least once a month/ week	3.60	1.03–12.60	0.05	0.72	0.42–1.25	0.25	0.47	0.16–1.39	0.17	0.82	0.53–1.26	0.35
Once in 3 months/ less	Ref.	Ref.	Ref.	Ref.	Ref.	Ref.	Ref.	Ref.	Ref.	Ref.	Ref.	Ref.
**Pregnancy complication**^**1**^												
Any	0.48	0.14–1.65	0.24	1.30	0.70–2.40	0.41	1.72	0.57–5.14	0.33	1.29	0.76–2.18	0.34
None	Ref.	Ref.	Ref.	Ref.	Ref.	Ref.	Ref	Ref.	Ref.	Ref.	Ref.	Ref.
**Transportation to delivery facility**												
Ambulance^2^	2.82	0.98–8.06	0.05	1.99	1.12–3.54	0.02	NA	NA	NA	2.02	1.22–3.33	0.01
Other	Ref.	Ref.	Ref.	Ref.	Ref.	Ref.				Ref.	Ref.	Ref.
**Delivery type**												
Normal	NA	NA	NA	Ref.	Ref.	Ref.	NA	NA	NA	Ref.	Ref.	Ref.
Other				1.63	0.66–4.01	0.29				1.67	0.70–3.96	0.24
**Delivery outcome**												
Single	NA	NA	NA	0.10	0.06–0.19	< 0.01	0.12	0.01–1.24	0.08	0.09	0.05–0.19	< 0.01
Multiple				Ref.	Ref.	Ref.	Ref.	Ref.	Ref.	Ref.	Ref.	Ref.
**Delivery complication**^**3**^												
Any	5.58	1.76–17.66	< 0.01	1.02	0.50–2.08	0.96	1.08	0.34–3.45	0.90	1.33	0.76–2.33	0.31
None	Ref.	Ref.	Ref.	Ref.	Ref.	Ref.	Ref.	Ref.	Ref.	Ref.	Ref.	Ref.
**Postnatal care either at home or at facility**												
Yes	2.53	0.62–10.32	0.20	1.08	0.61–1.91	0.80	1.50	0.51–4.44	0.46	1.36	0.80–2.29	0.26
No	Ref.	Ref.	Ref.	Ref.	Ref.	Ref.	Ref.	Ref.	Ref.	Ref.	Ref.	Ref.
**48 h facility stay post delivery**												
Yes	0.34	0.09–1.30	0.11	1.28	0.72–2.29	0.41	NA	NA	NA	0.98	0.58–1.63	0.92
No	Ref.	Ref.	Ref.	Ref.	Ref.	Ref.				Ref.	Ref.	Ref.
**Place of delivery**												
NM supported facilities										0.81	0.35–1.86	0.61
Facilities not supported by NM										0.84	0.36–1.97	0.69
Home deliveries										Ref	Ref.	Ref.
**Interaction between round and NM supported facilities**										0.27	0.07–1.13	0.07
Facilities not supported by NM										0.59	0.20–1.73	0.34
Home deliveries										Ref.	Ref.	Ref.

^1^Swelling of hands/feet and face, excessive vomiting/giddiness, headache/visual disturbances, weakness/ excessive fatigue, convulsions not with fever, weak or no movement of fetus, abnormal position of fetus, jaundice, vaginal bleeding, vaginal discharge, malaria, abdominal pain, preterm/ premature rupture of membrane, preterm labour

^2^Includes the Government-provided emergency transportation services

^3^Premature labour, preterm/premature rupture of membrane, prolonged labour (> 12 h), obstructed labour, breech/malpresentation

*Ref* Reference category

*NA* Not applicable

Among children born in NM facilities, the estimated HR for mortality at endline was significantly lower than at baseline [HR: 0.23, 95% CI: 0.06–0.82, *p* = 0.02]. This was not the case for children born at non-NM facilities. Factors other than survey round that were significantly associated with neonatal mortality among the NM facilities included: whether consumed 100 IFA tablets during pregnancy; whether met with an ASHA at least once a month during pregnancy; whether an ambulance was used to reach the delivery point; and whether the delivery had any complication. Among children born in non-NM supported facilities, factors that were independently associated with neonatal mortality included: whether received two doses of tetanus toxoid injections during pregnancy; whether an ambulance was used to reach the delivery point; and whether the delivery was singleton. In these facilities, the estimated hazard ratio at endline compared to baseline was not statistically significant. Among home deliveries, none of the risk factors considered were significantly associated with neonatal mortality, and the hazard ratio at endline compared to baseline was also not statistically significant.

## Discussion

Our findings suggest that the nurse mentoring program, which was designed to improve the quality of care for women and children during the labour, delivery and post-partum periods, was associated with a significant decline over time in neonatal mortality. There was a three-fold reduction in the neonatal mortality rate over a three-year period observed among deliveries in primary heath care facilities with NM programs, with the greatest reduction in mortality seen on the first day of life. This corresponds to an average annual rate of reduction in NMR of about 23%. No significant changes were observed in the estimated NMR among newborns delivered in facilities not supported by the NM program or at home. Differences between place of delivery groups were also observed in the late postnatal period, and no neonatal deaths were reported among infants who delivered in NM facilities from day 8–28 post-birth.

Analysis of known risk factors for neonatal death that may have acted as confounders suggests that they were unlikely to have accounted for the large change in neonatal mortality in our study, as there were no significant differences in socio-demographic characteristics between the two survey rounds. Interestingly, a greater proportion of more marginalized women utilized services at the NM PHCs, suggesting that the NM program was able to reach a population where a greater burden of neonatal mortality has been documented [17–19]. Significant improvements were seen in pregnancy-associated characteristics between survey rounds, but this was similar in both the NM and non-NM facility groups, and is similar to trends in utilization of health services from other studies [20, 21]. With the exception of an increase in Caesarean sections and assisted operative vaginal deliveries, there were also no significant differences in delivery characteristics between baseline and endline. Encouragingly, there was a significant improvement between the two survey rounds in the proportion of women who received postnatal care, either at home or in a facility (53 to 80%).

Although the study was not designed to establish causal linkages between the NM program and reductions in neonatal mortality, several aspects of the NM program could have contributed to the positive effect on newborn survival. These would include: a focus on improving diagnosis and management of MNCH complications through enhanced skills, knowledge and practices; use of self-check lists to identify system level gaps and develop action plans to respond appropriately; and use of case sheets as job aids to prompt appropriate treatment and referral to ensure follow-up once complications are identified. We have previously shown that this multipronged approach resulted in improved quality of care at institutions supported by the NM program within the project area, through improvements in facility readiness as well as provider preparedness, and improved knowledge and skills around essential obstetric and neonatal care [10, 11].

From 2009 to 2011, the average annual rate of reduction in NMR for all of India was 4·6% [22]. The reduction in NMR observed in our study was much higher than this, and several factors may have contributed to this finding. One is timing, as the greatest reduction in mortality observed in our program occurred on the first day, when in India more than one-third of newborn deaths occur [19, 22]. Inability to prevent early neonatal death is a known contributor to slow progress in newborn survival [22], and the nurse mentoring program was designed to address MNCH care during this critical period. Secondly, the context of being embedded in the Government of India’s NRHM likely facilitated the accelerated mortality decline, as the program built on gains already achieved by the NRHM. Demand for delivery in health facilities was already high in the state (although lower in our program districts), and key infrastructure, supplies, personnel and emergency transport were already in place when our nurse mentoring program began [23]. Furthermore, faster rates of decline in NMR have been documented in more marginalized and vulnerable populations [17, 24], and our data indicate that a large and increasing proportion of marginalized women utilized services at the health facilities where the nurse-mentoring program was implemented; and this may have also contributed to an accelerated rate of decline.

We also attempted to identify predictors of newborn survival for the overall sample population and sub-populations by place of delivery. When survey round and place of delivery were controlled for, single births and births to mothers who had received two tetanus toxoid injections during pregnancy were associated with increased newborn survival. This is not surprising, as multiple gestation is recognized as a risk factor for neonatal morbidity and mortality [25, 26], and the protective effect of maternal vaccination for TT on neonatal mortality in India is well-documented [27, 28].. Among newborns delivered in NM facilities, factors that were significantly associated with neonatal mortality included: consumption of 100 IFA tablets during pregnancy; meeting with an ASHA at least once a month during pregnancy; use of an ambulance to reach the delivery point; and presence of any delivery complication. We hypothesize that these factors may represent proxies for high-risk pregnancies, leading to adverse outcomes. Use of an ambulance, for example, may be due to a pregnancy or neonatal medical emergency. From 2012 to 2104, 40–43% of the medical emergency trips in Karnataka were pregnancy related [29]. A hospital-based study in Karnataka observed that one-fifth of the patients arriving in hospitals using government ambulance services had pregnancy-related complications [30].

Recently, the Government of India has made provisions for nurse mentors in several states and in high priority districts. For instance, the Government of India has been supporting, since 2014, a set of 100 on-site nurse mentors in 25 high priority districts of Uttar Pradesh, a large north Indian state. Various elements of the NM intervention were incorporated in the National Guidelines on Quality Assurance [31], strengthening competency-based training of health care providers [32], and strengthening pre-service education for nursing [33].

We used household surveys to determine neonatal mortality, which are often considered better sources of data on neonatal mortality than either the civil registration data or the routine facility data [8, 34]. The civil registration data is often incomplete [35]. Facility data are limited by substantial selection bias since births and deaths may occur outside the health system [36]. Data from household surveys where calculations are based on full birth history, date of birth of each child, whether each child is alive and if not the age of death are hence used for neonatal mortality estimates. In India, the Sample Registration System (SRS) and the household surveys have been the major sources of data on neonatal mortality. India has completed 4 rounds of National Family Health Survey (NFHS), designed and implemented on the models of Demographic and Health Surveys. And the SRS data on neonatal mortality has been made available since 1971. However, both the NFHS and the SRS do not provide the neonatal mortality estimates for the project area. And hence special surveys, at baseline and end line were designed and implemented, similar to the NFHS, in the project districts.

Our study has several limitations. We used a non-randomized design to compare the health outcomes between different types of facilities and home deliveries, and so we cannot directly attribute the observed decline in neonatal mortality to the NM program. Moreover, we have used population-level data to measure neonatal mortality, rather than facility-level data, and thus there are limitations in terms of assessing the effectiveness of the NM program, which is a facility-based intervention. The facilities have significant heterogeneity in terms of institutional capacity and numbers of complicated cases. While a randomized controlled trial among the PHCs would have been ideal, this was not possible due to the desire of the state government to institute rapid program scale-up. Nevertheless, we believe that the comparisons made between NMR at baseline and endline as a measure of program effect are suggestive, because participants in the surveys were randomly recruited; they had no information about the NM intervention per se, and thus had no bias for use of PHCs over non-PHCs as delivery points; and the NM intervention was implemented in rapid succession in all PHCs in the project districts, using similar processes and content. Another limitation is that we do not have information on referral pathways, so cannot know if those who delivered at non-PHC facilities were first seen or referred from NM PHCs. However, as higher-level institutions also showed some improvement in their NMR, it is unlikely that the rapid decline in NMR at PHCs was only due to referral of complicated cases, and shifting of place of newborn death.

## Conclusions

In summary, this study provides evidence supporting the linkage between quality improvement strategies and improved MNCH outcomes, evidence which to date has been lacking [12–14]. Findings from the study suggest that nurse mentoring may represent a successful strategy to decrease NMR when implemented in the correct context. Further research is required to prove its effectiveness, but if confirmed, this strategy should be helpful in guiding the development of quality improvement strategies in labour, delivery and newborn health care that are suitable for implementation and scale-up in settings with high neonatal mortality.

## Data Availability

The data are hosted on the CGPH PSDP server at the University of Manitoba, and a de-identified data cut will be made available on request from the CGPH PSDP Data Steward (contact e-mail: Nancy.Yu@umanitoba.ca).

## Abbreviations

NRHM: National Rural Health Mission
MNCH: Maternal, newborn and child health
NM: Nurse mentoring
SN: Staff nurses
ANM: Auxilliary Nurse Midwife
PHC: Primary Health Centre
ANC: Antenatal care
TT: Tetanus Toxoid
IFA: Iron and Folic Acid
ASHA: Accredited Social Health Activist
SC: Scheduled caste
ST: Scheduled tribe
NMR: Neonatal mortality rate
HR: Hazard Ratio

## References

[1] National Institute of Medical Statistics, Indian Council of Medical Research and UNICEF. Infant and Child Mortality in India: Levels, Trends and Determinants. India Country Office. New Delhi: National Institute of Medical Statistics, Indian Council of Medical Research and UNICEF; 2012. Available from: http://unicef.in/CkEditor/ck_Uploaded_Images/img_1365.pdf. Accessed 4 Mar 2017.

[2] Black RE, Levin C, Walker N, Chou D, Liu L, Temmerman M for the DCP3 RMNCH Authors Group (2016). Reproductive, maternal, newborn, and child health: key messages from Disease Control Priorities 3rd Edition. Lancet.

[3] Paul VK, Sachdev HS, Mavalankar D (2011). Reproductive health, and child health and nutrition in India: meeting the challenge. Lancet.

[4] Rammohan A, Iqbal K, Awofeso N. Reducing neonatal mortality in India: critical role of access to emergency obstetric care. PLoS One 2013; 8(3): e57244. https://doi.org/10.1371/journal.pone.0057244. 10.1371/journal.pone.0057244 PMC3609864 23544038

[5] Lawn JE, Blencowe H, Oza S (2014). Every newborn: progress, priorities, and potential beyond survival. Lancet.

[6] https://data.gov.in/catalog/neo-natal-mortality-rate-india. Accessed 5 Oct 2016.

[7] Regional disparities in the state of Karnataka. Available from: http://planning.kar.nic.in/sites/planning.kar.in/files/Dr_Nanjundappa/Ch_15_Health%20Infrastructure.pdf. Accessed 10 Feb 2017.

[8] Ram U, Jha P, Ram F (2013). Neonatal, 1–59 month, and under-5 mortality in 597 Indian districts, 2001 to 2012: estimates from national demographic and mortality surveys. Lancet Glob Health.

[9] http://pib.nic.in/newsite/PrintRelease.aspx?relid=118620. Accessed 6 Oct 2016.

[10] Jayanna K, Bradley J, Mony P (2016). Effectiveness of on-site nurse mentoring in improving quality of institutional births in the primary health Centres of high priority districts of Karnataka, South India: a cluster randomized trial. PLoS One.

[11] Bradley J, Jayanna K, Shaw S (2017). Improving the knowledge of labour and delivery nurses in India: a randomized controlled trial of mentoring and case sheets in primary care centres. BMC Health Serv Res.

[12] Jhpiego in India. Available from https://www.jhpiego.org/wp-content/uploads/2015/08/Jhpiego-India.pdf. Accessed 21 Nov 2016.

[13] Jayanna K (2013). Improving quality of care in maternal, newborn and child health: opportunities and challenges in India. Indian J Community Health.

[14] Dettrick Z, Firth S, Jimeniz SE. Do strategies to improve quality of maternal and child health care in lower and middle income countries lead to improved outcomes? A review of the evidence. PLoS ONE. 8(12):e83070. 10.1371/jpurnal.pone.0083070. 10.1371/journal.pone.0083070 PMC3857295 24349435

[15] Fischer EA, Jayanna K, Cunningham T, Washington M, Mony P, Bradley J, Moses S (2015). Nurse mentors to advance quality improvement in primary health Centres: lessons from a pilot program in northern Karnataka, India. Global Health Sci Pract.

[16] Målqvist M. Neonatal mortality: an invisible and marginalized trauma. Glob Health Action. 2011;4. 10.3402/gha.v4i0.5724. 10.3402/gha.v4i0.5724 PMC3060998 21423597

[17] Bendavid E (2014). Changes in child mortality over time across the wealth gradient in less-developed countries. Pediatr.

[18] McKinnon B, Kaufman JS, Bergevin Y (2014). Socioeconomic inequality in neonatal mortality in countries of low and middle income: a multicountry analysis. Lancet Global Health.

[19] Sankar MJ, Neogi NB, Sharma J (2016). State of newborn health in India. J Perinatol.

[20] Wenjuan W, Alva S, Wang S, Fort A (2011). Levels and trends in the use of maternal health Services in Developing Countries. DHS comparative reports no. 26.

[21] Hurst TE, Semrau K, Patna M, Gawande A, Hirschorn LR (2015). Demand-side interventions for maternal care: evidence of more use, not better outcomes. BMC Pregnancy Childbirth.

[22] Zodpey S, Paul VK, Public Health Foundation of India (2014). All India Institute of Medical Sciences and Save the Children. State of India’s Newborns (SOIN) 2014- a report.

[23] Ministry of Health and Family Welfare, Government of India (2011). Family Welfare Statistics in India 2011.

[24] Victora CG, Barros AJD, Franca GVA, da Silva ICM, Carvajal-Velez L, Amouzou A. The contribution of poor and rural populations to national trends in reproductive, maternal, newborn and child health coverage: analysis of cross-sectional surveys from 64 countries. Lancet Global Health 2017; published online Feb 23. http://dx.doi.org/10.1016/S2214-109x(17)30077-3. 10.1016/S2214-109X(17)30077-3 PMC5565524 28238719

[25] Vogel JP, Torloni MR, Seuc A, et al. Maternal and perinatal outcomes of twin pregnancy in 23 low-and middle-income countries. PLoS ONE 2013; 8(8): e70549.doic10.1371/journal.pone.0070549. 10.1371/journal.pone.0070549 PMC3731264 23936446

[26] Dudenhausen JW, Maier RF (2010). Perinatal problems in multiple births. Dtsch Arztebl Int.

[27] Singh A, Pallikadavath S, Ram F, Alagarajan M. Do antenatal care interventions improve neonatal survival in India? Health Policy Plan. 2013:1–7. 10.1093/heapol/czt066 PMC4471439 24038077

[28] Singh A, Kumar A, Kumar A (2013). Determinants of neonatal mortality in rural India, 2007-2008. Peer J.

[29] Raj AX. Saving lives through rural ambulance services: Experiences from Karnataka and Tamil Nadu states, India. Transport and Communications Bulletin for Asia and the Pacific, No. 84, 2014. Available from http://www.unescap.org/sites/default/files/Bulletin%2084_Article5.pdf. Accessed 27 Oct 2016.

[30] Jagadeesha HS (2011). Utilization of EMRI 108 service and associated factors in Bengaluru Urban District, Karnataka, India.

[31] Ministry of Health and Family Welfare (MOHFW) [India]. Operational guidelines for quality assurance in public health facilities. New Delhi: MOHFW; 2013. Available: http://www.rrcnes.gov.in/quality%20Assurance/Operational%20Guidelines%20on%20Quality%20Assurance%20%28Print%29.pdf. Accessed 24 Jan 2020.

[32] Ministry of Health and Family Welfare (MOHFW) [India]. Skills lab operational guidelines: strengthening competency based training of health care providers for reproductive maternal newborn child and adolescent health (RMNCH+A) services. New Delhi: MOHFW; 2013. Available: http://164.100.130.11:8091/training/SkillsLab_trainingManual.pdf. Accessed 24 Jan 2020.

[33] Ministry of Health and Family Welfare (MOHFW) [India]. Strengthening pre-service education for the nursing and midwifery cadre in India: operational guidelines. New Delhi: MOHFW; 2013. Available: http://nursingandmidwifery.gov.in/preservice/Operational%20Guideline-Final%20Compiled-Jan,%202013%202nd20edition.pdf. Accessed 24 Jan 2020.

[34] Oestergaard MZ, Inoue M, Yoshida S, Mahanani WR, Gore FM, et al. Neonatal mortality levels for 193 countries in 2009 with trends since 1990: a systematic analysis of Progress, projections, and priorities. PLoS Med. 2011;8(8):e1001080. 10.1371/journal.pmed.1001080, https://journals.plos.org/plosmedicine/article?id=10.1371/journal.pmed.1001080. Accessed 20 Jan 2020. 10.1371/journal.pmed.1001080 PMC3168874 21918640

[35] WHO https://www.who.int/healthinfo/civil_registration/en/. Accessed 20 Jan 2020.

[36] Measure Evaluation https://www.measureevaluation.org/rbf/indicator-collections/health-outcome-impact-indicators/neonatal-mortality-rate. Accessed 20 Jan 2020.

